# 3DupIC: An Underwater Scan Matching Method for Three-Dimensional Sonar Registration

**DOI:** 10.3390/s22103631

**Published:** 2022-05-10

**Authors:** António Ferreira, José Almeida, Alfredo Martins, Aníbal Matos, Eduardo Silva

**Affiliations:** 1 INESC TEC—Institute for Systems and Computer Engineering, Technology and Science, Rua Dr. Roberto Frias, 4200-465 Porto, Portugal; jose.m.almeida@inesctec.pt (J.A.); alfredo.martins@inesctec.pt (A.M.); anibal.matos@inesctec.pt (A.M.); eduardo.silva@inesctec.pt (E.S.); 2 ISEP—School of Engineering, Polytechnic Institute of Porto, Rua Dr. António Bernardino de Almeida, 431, 4249-015 Porto, Portugal; 3 FEUP—Faculty of Engineering, University of Porto, Rua Dr. Roberto Frias, 4200-465 Porto, Portugal

**Keywords:** probabilistic scan matching, underwater, AUV, Coda Echoscope 3D, ICP, registration, localization

## Abstract

This work presents a six degrees of freedom probabilistic scan matching method for registration of 3D underwater sonar scans. Unlike previous works, where local submaps are built to overcome measurement sparsity, our solution develops scan matching directly from the raw sonar data. Our method, based on the probabilistic Iterative Correspondence (pIC), takes measurement uncertainty into consideration while developing the registration procedure. A new probabilistic sensor model was developed to compute the uncertainty of each scan measurement individually. Initial displacement guesses are obtained from a probabilistic dead reckoning approach, also detailed in this document. Experiments, based on real data, demonstrate superior robustness and accuracy of our method with respect to the popular ICP algorithm. An improved trajectory is obtained by integration of scan matching updates in the localization data fusion algorithm, resulting in a substantial reduction of the original dead reckoning drift.

## 1. Introduction

The ability to accurately self localize is essential to achieve fully autonomous robotic agents. At the base level, most localization solutions incorporate a dead reckoning mechanism, where relative displacement measurements serve to incrementally update the localization estimate. This recursive strategy is characterized by unbounded uncertainty growth, resulting from successive integration of measurement errors. Hence, a periodic error reset is necessary to keep localization uncertainty within acceptable limits. This is usually accomplished by accessing some absolute positioning infrastructure, such as the Global Navigation Satellite System (GNSS), in the outdoors, and acoustic positioning networks underwater, or by sensing the surrounding environment through exteroceptive sensors. This last option is desirable, as it renders the robot self-sufficient and discards the need for external support infrastructures. In this article, we investigate the possibility to retrieve localization references from a 3D acoustic camera, by means of a scan matching procedure.

Given two range scans of the same scene, taken from different perspectives, scan matching is the process of computing the rigid body transformation that brings the scans together in the same reference frame. Assuming the sensor is fixed with respect to the robot frame, the transformation that best overlaps two scans corresponds to the relative robot displacement between scan acquisition poses. Hence, by registering consecutive scans, relative displacement measurements can be retrieved and used for dead reckoning, usually with better accuracy than odometry or inertial-based solutions. Moreover, whenever the robot revisits previously explored areas, scan matching can be attempted with respect to an old recorded scan, to perform loop closure and correct the accumulated localization error.

Despite the large body of work on scan matching, underwater applications have been barely covered, simply because underwater range sensors perform much worse than terrestrial ranging devices. Underwater mapping references come mainly from sonar, whose measurements lack resolution and are characterized by poor accuracy, low sampling rate, insufficient area coverage, substantial noise and significant outlier percentage.

Some of the limitations are surpassed in this work thanks to an exceptional 3D acoustic camera—the Echoscope 3D from Coda Octopus [1]—rarely available on board surveying AUVs, due to its high price and heavy weight. The Echoscope 3D is a high resolution profiling sonar capable of acquiring, from a single ping, a square 128 × 128 matrix of range measurements. The ability to observe a broad seafloor area ensures the possibility of capturing consecutive overlapping scans, making this device ideal for 3D scan matching.

Despite the remarkable measurement density, the Echoscope 3D is still a sonar-based sensor, whose measurements show substantial noise and angular uncertainty. Therefore, a new sensor model is applied to represent the uncertainty of each individual range measurement. Registration is accomplished with a new probabilistic scan matching variant, called 3D underwater probabilistic Iterative Correspondence (3DupIC), which takes both measurements and their uncertainty into consideration, to develop consistent data association and error minimization. Robot displacement estimates, between scan acquisition poses, are computed through a custom dead reckoning algorithm, and supplied as initial guesses to the 3DupIC. While other underwater implementations resort to submap building, the 3DupIC directly develops scan matching based on the raw sonar data. Moreover, our implementation develops scan matching in 6 degrees-of-freedom, allowing the correction of position and attitude localization states.

### Related Work

From the extensive literature on scan matching, the Iterative Closest Point (ICP) [2] stands out as the most popular algorithm. It operates directly on the raw scan points, recursively refining the relative transformation, by minimizing the Euclidean distance between point-to-point matches. Repeating the expensive pairwise matching step in every iteration constitutes a major speed bottleneck [3]. Moreover, convergence to the global minimum is not guaranteed and the method is susceptible to outliers. Convergence becomes harder as sensor rotation increases, since the Euclidean distance metric does not capture the rotational component conveniently. Therefore, countless variants of the ICP algorithm have been proposed [4], either to increase speed [5] or improve robustness, convergence and accuracy [4,6,7,8].

Nevertheless, point-to-point correspondences give ICP the ability to generalize well over different environments, thus early attempts were made to use ICP for pairwise registration of 3D underwater scans [9,10], captured with an Echoscope acoustic camera [1], similar to the sensor used in the present work. Despite the highly structured testing environment and the substantial overlapping between consecutive scans, an outlier rejection stage was found to be essential to achieve convergence.

According to the author’s knowledge, there were no further developments on underwater scan matching using the Echoscope 3D until now. Nonetheless, several studies pursued a similar strategy of registering sonar scans using ICP, but this time focusing on sparse ultrasonic devices, both indoors [11] and underwater [12]. The sparse nature of single-beam sonar data denies the immediate application of scan matching, as information captured in a given instant provides insufficient information about the structure of the surrounding environment. As an alternative, both [11,12,13] resort to a submap-forming strategy, where sensor data, gathered for some period of time, are used to form a denser representation by grouping consecutive measurements based on dead reckoning.

However, sparsity is not the only weakness associated with sonar, and while modelling individual range measurements as point-like particles provides a plausible approximation for high-resolution terrestrial laser scanners, applying the same principle to noisier sonar references certainly constitutes a simplistic and overconfident interpretation of the data. Even when dealing with indoor laser scans, the work from Pfister et al. [14] has shown that careful error modelling greatly improves convergence and accuracy. Following the same premise, Montesano et al. [15] presented a new scan matching method, which takes noise into consideration in every phase of the algorithm—the probabilistic Iterative Correspondence (pIC). Here, scan points are modelled as Gaussian random variables and the Mahalanobis distance establishes the matching criteria to find statistically compatible point associations between scans. From the set of compatible points, a virtual association point is computed, in an effort to encapsulate all information provided by the set of compatible points. The squared Mahalanobis distance serves as cost function to minimize the point matching errors, through non-linear least squares. As opposed to the Euclidean distance, adopted by the traditional ICP, the Mahalanobis distance conveniently captures rotation information, making pIC more robust to sensor rotation. Experiments based on real laser scan data demonstrated the pIC’s superiority over standard scan matching techniques, converging more often, with higher accuracy and requiring less iterations [15].

Soon after, variants of the pIC have been developed for 2D scan matching with ultrasonic sensors, namely the sonar probabilistic Iterative Correspondence (spIC) [16], for indoor scenarios, and underwater implementations such as the MSISpIC [17] and the uspIC [18], both using mechanical scanning imaging sonars (MSIS). Given the sparse nature of sonar measurements all these techniques incorporate a submap building strategy, whose formulation constitutes the main aspect of differentiation between them. In a direct comparison [18], using the same data set, the uspIC outperforms the MSISpIC due to several improvements, such as, choosing a central submap origin to achieve a more balanced uncertainty distribution; adopting the Individual Compatibility Nearest Neighbor (ICNN) for data association, which is preferable when dealing with noisy sonar readings [19]; and using the scan matching result to correct the dead reckoning drift, accumulated during the submap forming process. The localization accuracy can be further enhanced by integrating probabilistic scan matching into Simultaneous Localization and Mapping (SLAM) solutions [20,21,22,23].

Subsequent improvements on data association were presented in [24], introducing a coarse registration, based on Monte Carlo pose sampling, for quick computation of better initial guesses for the robot displacement, in an effort to speed up and facilitate convergence. In a second stage, a fine alignment is accomplished based on a point-to-plane data association, known for performing better for small misalignments. Further, a grid-based search, which takes point uncertainty into consideration, is employed to reduce the matching effort from quadratic to linear complexity.

Unlike MSIS devices, that scan along the horizontal plane, multibeam profiling sonars are usually pointed down, to acquire swaths of the seabed, whose morphology is mostly flat and featureless. For such cases, Palomer et al. [25,26] propose an adaptive point sampling, based on an octree, with the objective of removing less informative points laying on flat areas, while preserving points in zones with a more salient structure. Despite the fact that involving less points in scan matching helps to reduce computational time, no substantial advantages, in terms of accuracy and convergence, were verified [25].

The literature shows that probabilistic scan matching techniques can effectively perform well in underwater environments based on noisy sonar data [17,18,20,21,22,23]. In addition to improving the mapping consistency, updates retrieved from probabilistic scan matching can also be exploited to reduce localization drift. However, it does not mean a probabilistic scan matching method will always improve dead reckoning, especially when the dead reckoning solution is of high quality, as reported in [25]. The full potential of this technique is reached when integrated in a SLAM solution, where probabilistic scan matching can be used to close loops and bound the overall localization uncertainty.

This paper is organized as follows. Section 2 presents the 3DupIC scan matching method, starting by detailing the probabilistic model for a single ultrasonic beam. Individual beams are later combined to form the probabilistic scan model. The dead reckoning approach, to retrieve initial registration guesses, is also presented. Finally, the complete 3DupIC algorithm is described. Results are presented in Section 3, where the 3DupIC is evaluated and compared with the ICP algorithm. Finally, Section 4 recapitulates the main contributions and most important findings and establishes future developments.

## 2. The 3D Underwater Probabilistic Iterative Correspondence

The 3DupIC is a probabilistic scan matching method, which adapts the original pIC [15], to perform registration, in six degrees of freedom, of 3D scans acquired from an Echoscope 3D underwater sonar. With this purpose in mind, a dedicated probabilistic sensor model was developed, to conveniently represent the main sources of measurement uncertainty. In order to track the robot’s movement in between scan acquisitions, a dead reckoning solution, based on the Extended Kalman Filter (EKF), was also developed.

### 2.1. Probabilistic Sonar Modelling

**Figure 1:**
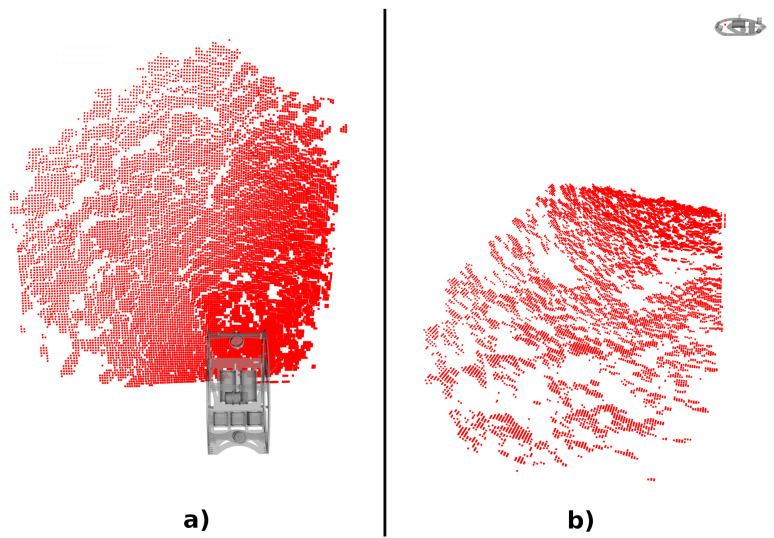
Illustration of a point cloud obtained from a single ping of the Echoscope 3D, where (**a**) is a top view and (**b**) a side view of the same scan. The scan is composed of a matrix of 128×128 points, represented in red. The robot model, in grey, represents the scan acquisition pose.

An Echoscope 3D scan is composed of readings arranged in a 128 by 128 matrix, as illustrated in Figure 1. First, we derive the model for each scan point individually, adopting a conic beam-shaped ping, commonly associated with profiling sonars. Finally, all independent beam models are placed in the sensor reference frame, to produce the probabilistic model for the entire scan.

#### 2.1.1. Individual Beam Model

**Figure 2:**
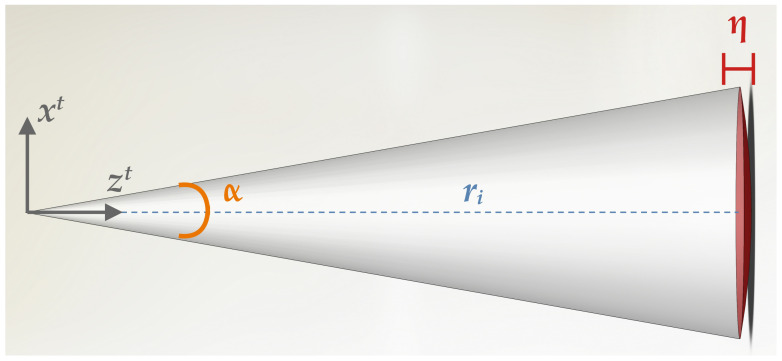
Illustration of the probabilistic beam model adopted by the 3DupIC to represent each single scan point.

Considering the conic beam model in Figure 2, two main sources of uncertainty arise: the angular beam aperture α originates a target area, which grows with respect to the slant range *r*, introducing ambiguity in the plane normal to the beam direction; on the other hand, the intrinsic sensor range resolution η comprises the main source of measurement uncertainty along the beam direction. The 3DupIC takes these parameters into account to model each individual sonar beam *i*, in the beam reference frame (*t*), as a Gaussian random vector: $b_{i}^{t}=N\left({\hat{b}}_{i}^{t},\;\Sigma_{i}^{t}\right)$. The mean ${\hat{b}}_{i}^{t}$ is directly defined by the measured range $r_{i}$, along the Z-axis: ${\hat{b}}_{i}^{t}={\left[0,0,r_{i}\right]}^{T}$. In the beam reference frame, with the Z-axis passing through the center of the cone, as illustrated in Figure 2, the *i*th beam uncertainty is characterized by the following covariance matrix:

(1) $$\Sigma_{i}^{t}=\left[\begin{array}{ccc} {\left(r_{i}\cdot \tan\left(\alpha \;/\;2\right)\right)}^{2} & 0 & 0\\ 0 & {\left(r_{i}\cdot \tan\left(\alpha \;/\;2\right)\right)}^{2} & 0\\ 0 & 0 & {\left(\eta \;/\;2\right)}^{2} \end{array}\right]$$

The first two diagonal elements of the covariance matrix characterize the uncertainty along the horizontal plane, associated with the size of the insonified area. This area takes a circular shape, with standard deviation given by the radius of the beam’s cone base $\left(r_{i}\cdot \tan\left(\alpha \;/\;2\right)\right)$. The third diagonal element characterizes the uncertainly along the beam direction, with a standard deviation equal to half the range resolution.

#### 2.1.2. Complete Scan Modeling

Now, let us consider a complete scan, provided by the sensor as a collection of *n* three-dimensional points $\left\{p_{1}^{s},\cdots ,p_{n}^{s}\right\}$, defined in the sensor reference frame (*s*). The probabilistic scan model consists of a collection of individual beams $S_{new}^{s}=\left\{b_{1}^{s},\ldots ,b_{n}^{s}\right\}$, each one represented by a multivariate Gaussian distribution $b_{i}^{s}=N\left({\hat{b}}_{i}^{s},\;\Sigma_{i}^{s}\right)$, centered on the sonar measurement (${\hat{b}}_{i}^{s}=p_{i}^{s}$), with covariance matrix given by:

(2) $$\Sigma_{i}^{s}=R_{t,i}^{s}\Sigma_{i}^{t}{\left(R_{t,i}^{s}\right)}^{T}$$

where $R_{t,i}^{s}$ is a rotation matrix, from the beam reference frame (*t*) to the sensor reference frame (*s*), used to transform the *i*th beam covariance matrix $\Sigma_{i}^{t}$. This rotation is first formulated using a quaternion and later converted into the rotation matrix $R_{t,i}^{s}$. The method starts by computing vector ${\vec{v}}_{q}$, normal to the plane defined by vectors ${\vec{p}}_{i}^{s}$ and ${\vec{v}}_{z}$, using the cross product:

(3) $${\vec{v}}_{q}={\vec{p}}_{i}^{s}\times {\vec{v}}_{z}$$

where × corresponds to the cross product operation and ${\vec{v}}_{z}$ is a direction vector aligned with the Z-axis of the sensor reference frame. The beam covariance matrix must rotate around vector ${\vec{v}}_{q}$ with an angle of $\alpha_{q}$, given by:

(4) $$\alpha_{q}=-\arccos\frac{{\vec{p}}_{i}^{s}\cdot {\vec{p}}_{z}}{∥{\vec{p}}_{i}^{s}∥∥{\vec{p}}_{z}∥}$$

where ${\vec{p}}_{i}^{s}\cdot {\vec{p}}_{z}$ indicates the dot product between vectors ${\vec{p}}_{i}^{s}$ and ${\vec{p}}_{z}$. From the direction vector ${\vec{v}}_{q}$ and the angle $\alpha_{q}$, the quaternion $q_{i}^{s}$ is constructed:

(5) qis=q0q1q2q3=cos(αq/2)v→qsin(αq/2)

The rotation matrix is finally obtained from the quaternion:

(6) $$R_{i}^{s}=\left[\begin{array}{ccc} q_{0}^{2}+q_{1}^{2}-q_{2}^{2}-q_{3}^{2} & 2q_{1}q_{2}-2q_{0}q_{3} & 2q_{1}q_{3}+2q_{0}q_{2}\\ 2q_{1}q_{2}+2q_{0}q_{3} & q_{0}^{2}-q_{1}^{2}+q_{2}^{2}-q_{3}^{2} & 2q_{2}q_{3}-2q_{0}q_{1}\\ 2q_{1}q_{3}-2q_{0}q_{2} & 2q_{2}q_{3}+2q_{0}q_{1} & q_{0}^{2}-q_{1}^{2}-q_{2}^{2}+q_{3}^{2} \end{array}\right]$$

Since all beam directions in the sensor array remain constant between scans, the calculation of this rotation matrix is only performed once, as soon as the first valid measurement for the *i*th beam arrives.

#### 2.1.3. Reference Frame Transformations

During the registration process, the scan needs to be converted to other reference frames, particularly to the navigation (n) frame, which implies passing through the intermediate body reference frame (b). The multivariate Gaussian distributions, with mean ${\hat{b}}_{i}^{n}$ and covariance $\Sigma_{i}^{n}$, for the *i*th beam, in the navigation reference frame are obtained as follows:

(7) $${\hat{b}}_{i}^{n}=R_{b}^{n}\underset{{\hat{b}}_{i}^{b}}{\underbrace{\left(R_{s}^{b}{\hat{b}}_{i}^{s}+t_{s}^{b}\right)}}+t_{b}^{n}$$

(8) $$\Sigma_{i}^{n}=R_{b}^{n}\underset{\Sigma_{i}^{b}}{\underbrace{R_{s}^{b}\Sigma_{i}^{s}{\left(R_{s}^{b}\right)}^{T}}}{\left(R_{b}^{n}\right)}^{T}$$

where $R_{s}^{b}$ and $t_{s}^{b}$ define, respectively, the rotation matrix and translation vector from the sensor to the body reference frame. These parameters, obtained through a previous calibration procedure, allow the beam to be transformed to the body frame $b_{i}^{b}=N\left({\hat{b}}_{i}^{b},\;\Sigma_{i}^{b}\right)$. The transformation from the body to the navigation frame, encoded by the rotation matrix $R_{b}^{n}$ and the translation vector $t_{b}^{n}$, represents the robot localization estimate, determined by the dead reckoning method presented next.

### 2.2. Dead Reckoning Localization

To facilitate convergence, an initial guess of the robot displacement should be supplied to the scan matching method. Moreover, the 3DupIC takes the uncertainty about the robot localization into consideration to develop the probabilistic registration. Therefore, a probabilistic localization strategy was implemented, to compute the robot pose and the underlying uncertainty, using the Extended Kalman Filter (EKF). The system receives angular velocity measurements as input, from a triad of fiber optic gyroscopes (FOG), plus linear velocity observations from a Doppler Velocity Log (DVL). The state vector, with 9 states ${\hat{x}}_{k}={\left[{\hat{p}}_{k}^{n},\;{\hat{\alpha }}_{k}^{n},\;{\hat{\nu }}_{k}^{n}\right]}^{T}$, represents position ${\hat{p}}_{k}^{n}=\left[{\hat{x}}_{k}^{n},\;{\hat{y}}_{k}^{n},\;{\hat{z}}_{k}^{n}\right]$; orientation, using Euler angles, ${\hat{\alpha }}_{k}^{n}=\left[{\hat{\phi }}_{k}^{n},\;{\hat{\theta }}_{k}^{n},\;{\hat{\psi }}_{k}^{n}\right]$ and velocity in the navigation reference frame ${\hat{\nu }}_{k}^{n}=\left[{\hat{u}}_{k}^{n},\;{\hat{v}}_{k}^{n},\;{\hat{w}}_{k}^{n}\right]$. Inertial measurements are integrated in the prediction step, to compute the robot’s orientation, while velocity observations are fused in the update step.

#### 2.2.1. State Prediction

The state prediction involves a constant velocity model to compute velocity and position, while orientation states are predicted using a simple inertial mechanization model:

(9) $${\hat{x}}_{k}=f\left({\hat{x}}_{k-1}\right)=\left[\begin{array}{c} {\hat{p}}_{k}^{n}\\ {\hat{\alpha }}_{k}^{n}\\ {\hat{\nu }}_{k}^{n} \end{array}\right]=\left[\begin{array}{c} {\hat{p}}_{k-1}^{n}+{\hat{\nu }}_{k-1}^{n}\Delta t\\ {\hat{\alpha }}_{k-1}^{n}+E_{b}^{n}\omega^{b}\Delta t\\ {\hat{\nu }}_{k-1}^{n} \end{array}\right]$$

where the matrix $E_{b}^{n}$ converts the angular velocities $\omega^{b}$, measured by the gyroscopes in the body frame, into the Euler angles rate of change:

(10) $$E_{b}^{n}=\left[\begin{array}{ccc} 1 & \sin\left({\hat{\phi }}_{k-1}^{n}\right)\tan\left({\hat{\theta }}_{k-1}^{n}\right) & \cos\left({\hat{\phi }}_{k-1}^{n}\right)\tan\left({\hat{\theta }}_{k-1}^{n}\right)\\ 0 & \cos\left({\hat{\phi }}_{k-1}^{n}\right) & -\sin\left({\hat{\phi }}_{k-1}^{n}\right)\\ 0 & \sin\left({\hat{\phi }}_{k-1}^{n}\right)\sec\left({\hat{\theta }}_{k-1}^{n}\right) & \cos\left({\hat{\phi }}_{k-1}^{n}\right)\sec\left({\hat{\theta }}_{k-1}^{n}\right) \end{array}\right]$$

The covariance matrix is computed through:

(11) $$P_{k}=F_{k}P_{k-1}F_{k}^{T}+Q_{k}$$

where the diagonal covariance matrix $Q_{k}$ characterizes the noise associated with the propagation of each state individually, and the matrix $F_{k}$ is the Jacobian of the motion model $f({\hat{x}}_{k-1})$ with respect to the localization states:

(12) Fk=∂p^kn∂p^kn∂p^kn∂α^kn∂p^kn∂ν^kn0∂α^kn∂p^kn∂α^kn∂α^kn∂α^kn∂ν^kn∂ν^kn∂p^kn∂ν^kn∂α^kn∂ν^kn∂ν^kn=I3×303×303×303×3∂α^kn∂α^kn03×303×303×3I3×3

where $I_{3\times 3}$ represents a 3 by 3 identity matrix and $0_{3\times 3}$ is a 3 by 3 null matrix. To prevent correlation between velocity and position states, a slight modification is performed in the top right corner, discarding the Jacobian expression.

#### 2.2.2. State Update

Before fusing the DVL measurements into the EKF, the measured velocities $\nu_{DVL}^{b}={\left[u_{DVL}^{b},v_{DVL}^{b},w_{DVL}^{b}\right]}^{T}$, in the body frame, are transformed into the robot velocities in the navigation reference frame $\nu_{DVL}^{n}={\left[u_{DVL}^{n},v_{DVL}^{n},w_{DVL}^{n}\right]}^{T}$, using the current robot orientation:

(13) $$\nu^{n}=R_{b}^{n}\cdot \nu^{b}$$

In this way, a direct velocity observation is produced: $z_{DVL}={\left[u_{DVL}^{n},v_{DVL}^{n},w_{DVL}^{n}\right]}^{T}$. Hence, the observation matrix **H** becomes:

(14) $$H=\left[\begin{array}{ccccccccc} 0 & 0 & 0 & 0 & 0 & 0 & 1 & 0 & 0\\ 0 & 0 & 0 & 0 & 0 & 0 & 0 & 1 & 0\\ 0 & 0 & 0 & 0 & 0 & 0 & 0 & 0 & 1 \end{array}\right]$$

The computation of the update state and covariance is performed according to the standard EKF expressions.

By performing the reference frame conversion on velocity outside the EKF framework, the presented formulation prevents correlations to form between velocity and orientation states. Furthermore, in the prediction step, correlation between velocity and position is avoided. If this care is disregarded, the position and orientation will become cross-correlated with velocity states, thus, through the update step, robot position and orientation would be indirectly corrected. This is not desirable, as the nature of dead reckoning presupposes that localization uncertainty increases monotonically.

The scan matching method starts by establishing the point correspondences between the reference scan $S_{\mathrm{ref}}=\left\{r_{1},\ldots ,r_{i}\right\}$, composed of *i* points, and the *j* points from the target scan $S_{\mathrm{target}}=\left\{t_{1},\ldots ,t_{j}\right\}$. The reference scan is defined in the global navigation frame.

### 2.3. Probabilistic Scan Matching

Let us consider a reference scan $S_{ref}^{n}=\left\{r_{1}^{n},\ldots ,r_{m}^{n}\;|\;r_{j}^{n}=N\left({\hat{r}}_{j}^{n},\;\Sigma_{j}^{n}\right)\right\}$, composed of *m* measurements, modeled according to our probabilistic sensor model and defined in the navigation reference frame. Eventually, the robot moves and a new scan is acquired. Let us refer to this new scan as the target scan $S_{target}^{s}=\left\{t_{1}^{s},\ldots ,t_{o}^{s}\;|\;t_{i}^{s}=N\left({\hat{t}}_{i}^{s},\;\Sigma_{i}^{s}\right)\right\}$, with *o* measurements, defined in the sensor reference frame.

Ideally, compounding each point of the new scan with the localization estimate, following Equation (7), produces a perfect registration. Unfortunately, in reality, several sources of uncertainty such as localization error, scan noise and wrong reference frame calibrations, lead to an inaccurate result.

The 3DupIC aims to enhance the registration consistency and at the same time retrieve localization corrections to improve the dead reckoning solution. The method evolves in two steps. First, for each point in the target scan, the most statistically close point in the reference scan is identified. By repeating the same process for every point, a set of scan matches is constructed. In a second stage, the optimization procedure refines the transformation, by minimizing distances between the matched point pairs. Both steps repeat until convergence.

#### 2.3.1. Finding Compatible Points

For each point $t_{i}^{s}$ in the target scan, all points in $S_{ref}^{n}$ are tested for similarity using the squared Mahalanobis distance:

(15) $$d_{ij}^{2}=\varrho_{ij}^{T}\Sigma_{ij}^{-1}\varrho_{ij}$$

where:

(16) $$\varrho_{ij}=f\left({\hat{x}}_{k},{\hat{t}}_{i}^{s}\right)-{\hat{r}}_{j}^{n}$$

represents the error between the *j*th reference point and the *i*th target point, this last one projected into the navigation frame, through function $f\left({\hat{x}}_{k},{\hat{t}}_{i}^{s}\right)$, defined in (Equation (7)). The covariance matrix $\Sigma_{ij}$, characterizing the matching uncertainty, is calculated using Equation (17).

(17) $$\Sigma_{ij}=\Sigma_{j}^{n}+\Sigma_{i}^{n}+J_{x}\left({\hat{x}}_{k},{\hat{t}}_{i}^{b}\right)P_{k}{\left[J_{x}\left({\hat{x}}_{k},{\hat{t}}_{i}^{b}\right)\right]}^{T}$$

The covariance matrices $\Sigma_{j}^{n}$ and $\Sigma_{j}^{n}$ encode the uncertainties of the scan point pairs, computed using Equation (8). Since localization uncertainty affects the matching result, its contribution to the matching uncertainty is computed by the expression $J_{x}\left({\hat{x}}_{k},{\hat{t}}_{i}^{b}\right)P_{k}$$\left[J_{x}\left({\hat{x}}_{k},{\hat{t}}_{i}^{b}\right)\right]$, where the matrix $J_{x}\left({\hat{x}}_{k},{\hat{t}}_{i}^{b}\right)$ is the Jacobian of the error function (Equation (16)) with respect to the localization states, evaluated at the current robot location ${\hat{x}}_{k}$ and at the scan point in the body frame ${\hat{t}}_{i}^{b}$. The matrix $P_{k}$ refers to the localization covariance matrix.

The Mahalanobis distance is assumed to be Gaussian, therefore, the squared Mahalanobis distance follows a chi-squared distribution $\chi_{q}^{2}$, where *q* is the dimensionality of vector $\varrho_{ij}$, in this case 3. A point $r_{j}^{n}$ from the reference scan is statistically compatible with a point $t_{i}^{s}$ if it passes the chi-squared test, i.e., the squared Mahalanobis distance is less than the inverse chi-squared cumulative function $\chi_{q\alpha }^{2}$, evaluated for a given confidence level α. Additionally, from the set of compatible points, only the one with a smaller Mahalanobis distance is selected to form the match pair with $t_{i}^{s}$. A point pair is defined as $<r_{j}^{n},t_{i}^{s}>:r_{j}^{n}=\arg\min d_{ij}^{2}\wedge d_{ij}^{2}<\chi_{n\alpha }^{2}$.

By repeating this search for all points in the target scan, hopefully, in case of sufficient scan overlap, a set of point matches $M=\{<r_{\kappa 1}^{n},t_{\kappa 1}^{s}>,\ldots ,<r_{\kappa n}^{n},t_{\kappa n}^{s}>\}$ is computed, where, for simplicity, κi represents the index pairing for the *i*th correspondence.

#### 2.3.2. Optimization

The optimization step aims to refine the robot displacement by minimizing the squared Mahalanobis distances between corresponding points:

(18) $${\hat{x}}_{3DupIC}=\min\sum \varrho_{\kappa i}^{t}\Sigma_{\kappa i}^{-1}\varrho_{\kappa i}.$$

Fortunately, Equation (18) has a closed-form solution given by:

(19) $${\hat{x}}_{3DupIC}={\left(J^{T}QJ\right)}^{-1}J^{T}QA$$

where:

(20) $$J=\left[\begin{array}{c} J_{x}\left({\hat{x}}_{k},{\hat{t}}_{\kappa 1}^{s}\right)\\ J_{x}\left({\hat{x}}_{k},{\hat{t}}_{\kappa 2}^{s}\right)\\ \vdots \\ J_{x}\left({\hat{x}}_{k},{\hat{t}}_{\kappa n}^{s}\right) \end{array}\right],\;A=\left[\begin{array}{c} J_{x}\left({\hat{x}}_{k},{\hat{t}}_{\kappa 1}^{s}\right){\hat{x}}_{k}-\varrho_{\kappa 1}\\ J_{x}\left({\hat{x}}_{k},{\hat{t}}_{\kappa 2}^{s}\right){\hat{x}}_{k}-\varrho_{\kappa 1}\\ \vdots \\ J_{x}\left({\hat{x}}_{k},{\hat{t}}_{\kappa n}^{s}\right){\hat{x}}_{k}-\varrho_{\kappa n} \end{array}\right]$$

and the matrix Q is a block diagonal matrix, containing the inverse of the covariance matrices (Equation (17)), characterizing the uncertainty of each pair:

(21) $$Q=\left[\begin{array}{cccc} {\left(\Sigma_{\kappa 1}\right)}^{-1} & \; & & \\ \; & {\left(\Sigma_{\kappa 2}\right)}^{-1} & \; & \\ \; & \; & \ddots & \\ \; & \; & & {\left(\Sigma_{\kappa n}\right)}^{-1} \end{array}\right]$$

## 3. Experimental Results

Due to the high computational complexity, the 3DupIC was coded in C++ and OpenCL, with most of the scan matching algorithm, including point matching and optimization, running on a Graphics Processing Unit (GPU). The achieved processing performance is satisfactory for offline testing, but still too slow for real time execution—a limitation to be addressed in the future. The tests presented in this article focus on assessing the accuracy and convergence of our probabilistic scan matching method.

To evaluate the 3DupIC’s performance, several tests were developed, based on real data collected during the field trials of the ¡VAMOS! project, at the Silvermines flooded quarry, in the Republic of Ireland. The dataset was acquired by the EVA AUV, equipped with an Echoscope 3D, plus a set of localization sensors, including DVL, IMU, pressure sensor, dual-antenna GNSS system [27] and acoustic positioning. In real time, a data fusion localization solution, detailed in [28], fuses all sensor information to obtain an accurate localization solution, used here as ground truth reference. In order to verify the advantage of our probabilistic method, results are also compared with the standard ICP method. For this purpose, the ICP implementation from the point cloud library (PCL) [29] was employed. Additionally, an outlier rejection stage, based on Random Sample Consensus (RANSAC), was applied to filter wrong nearest neighbor correspondences before each ICP optimization.

### 3.1. Initial Displacement Perturbation Experiment

**Figure 3:**
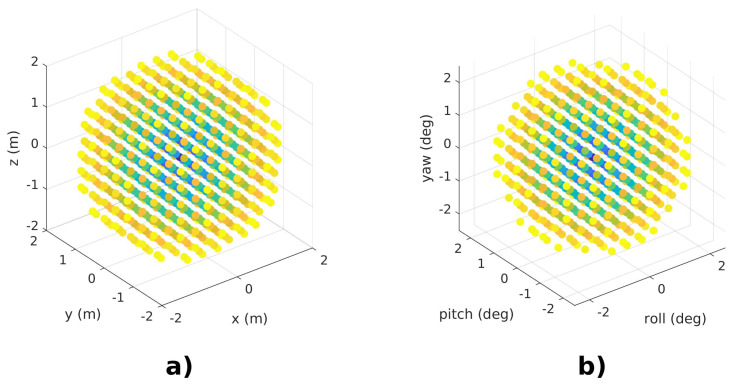
Initial perturbations introduced to (**a**) position states and (**b**) orientation states. Points are colored according to their Euclidean distance with respect to the true displacement, ranging from short distances, marked with blue, to higher distances in yellow.

The first test validates the convergence of both methods in the presence of initial localization errors. Here, two identical scans are registered together, repeating the experiment for different initial displacement errors. The initial errors result from uniform sampling, with increments of ±0.4 m, around the true pose, up to a maximum Euclidean distance of 2.25 m. Orientation errors were computed in a similar way, with increments of ±0.5 degrees, until the Euclidean distance of the angular errors reaches 2.5 degrees. Figure 3 shows the set of initial errors induced in this experiment.

By registering one scan with itself, the impact of the localization error is eliminated, as both scans are acquired from the same robot pose. Moreover, the reference and target scans are equally affected by noise, making the supplied displacement perturbation the dominant error source. Therefore, the registration algorithm should converge to the global minimum, which in this case is the transformation corresponding to the initial induced error. Both registration methods run until the Euclidean position, taken between the solutions of two consecutive iterations, falls below $2\times 10^{-4}$ m.

**Figure 4:**
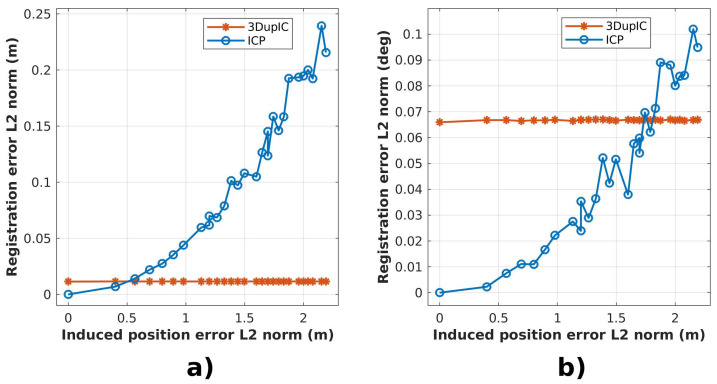
Relation between the final registration errors, expressed through the Euclidean distance of (**a**) position and (**b**) orientation registration errors, with respect to the norm of the position disturbance introduced initially to each registration method. The vertical axis corresponds to the average registration error, computed from the experiments executed for each sampled disturbance distance.

Initial perturbations introduced in the position states, cause the final registration errors depicted in Figure 4, expressed by the Euclidean distance between position and orientation states. It can be observed that the 3DupIC produces the most consistent results, always converging to a similar solution, while the ICP starts by providing more accurate results, but soon degrades in performance as the displacement error increases. For small initial disturbances, the ICP method outperforms the 3DupIC. This behavior may result from the localization covariance matrix supplied to the 3DupIC, which sets a high uncertainty margin, to encompass the maximum initialization error. Since the covariance matrix is kept constant during the test, it provides an underconfident uncertainty representation for smaller initial perturbations, which could explain the 3DupIC’s inability to respond as accurately as the ICP in those situations. This demonstrates that a balanced uncertainty representation is essential for the 3DupIC to develop consistent data association and error minimization.

**Figure 5:**
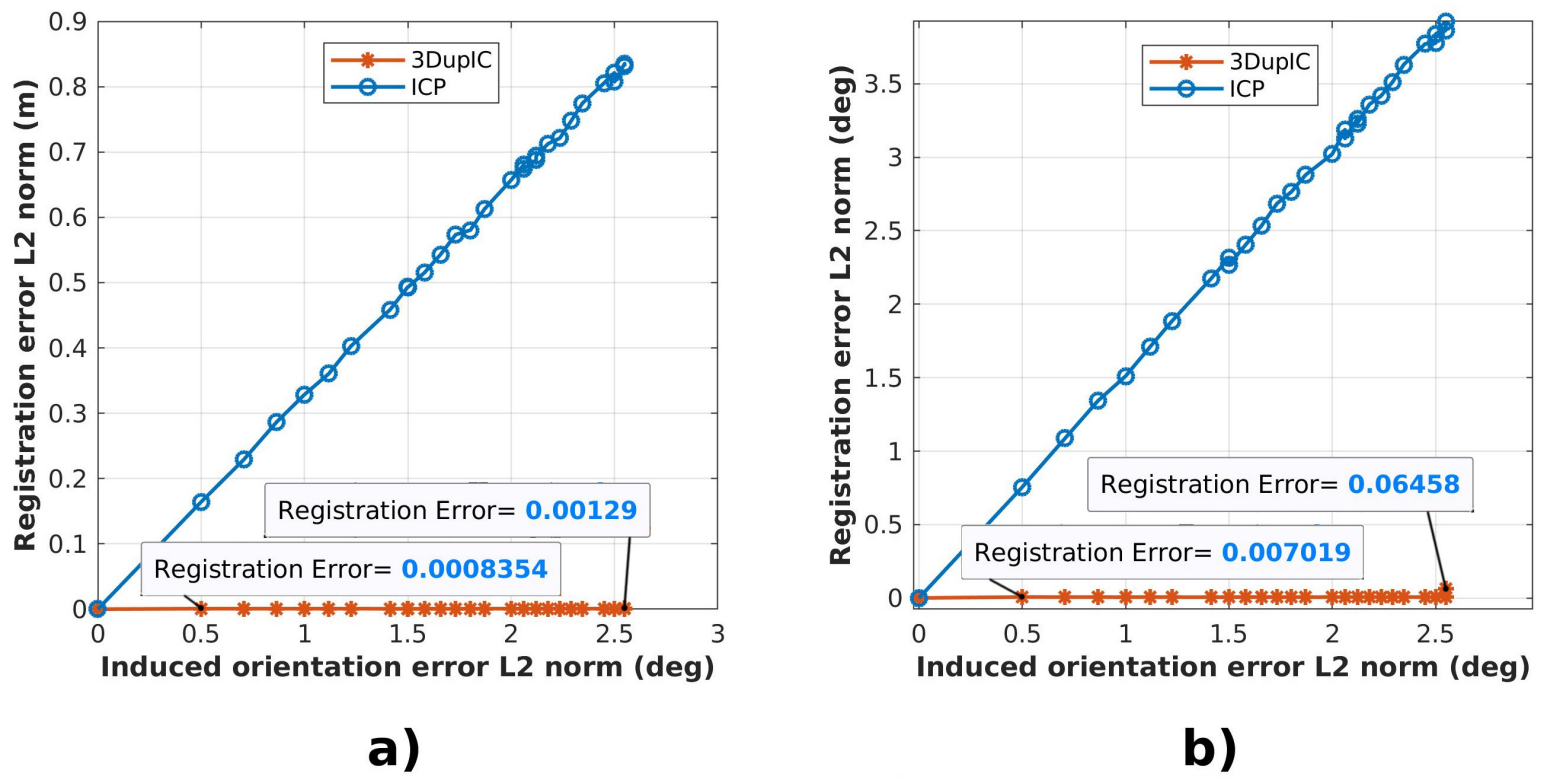
Induced errors in the initial orientation cause (**a**) position and (**b**) orientation registration errors, with respect to angular disturbance, represented in the horizontal axis. The vertical axis corresponds to the average registration error, computed from the experiments executed for each corresponding sampled disturbance distance.

A clear difference is noticed when initial orientation errors are introduced (Figure 5). Once again, the 3DupIC consistently provides an accurate solution, whereas the ICP’s performance rapidly deteriorates as result of poor orientation initialization. This observation verifies the difficulty of the ICP algorithm to respond appropriately to relative scan misalignment. In this particular case we can argue that the ICP method diverges, since the registration error in Figure 5b is higher than the induced orientation error. Moreover, it can be concluded that by relying on the Mahalanobis distance, the 3DupIC effectively captures the orientation component conveniently.

### 3.2. Short Trajectory Test

In the previous experiment, the same scan was registered with itself in an effort to eliminate the impact of scan and localization inaccuracies. However, in regular applications, scans do not overlap completely, due to robot motion and noise affecting each scan differently. In order to compare the ICP and 3DupIC methods in a more realistic scenario, different scan pairs are registered along a short robot trajectory. This time, the first acquired scan serves as reference, and all subsequent scans, obtained as the robot moves away, are registered with respect to this first reference scan. In this way, as the robot progresses its mission moving forward, less overlap between scans is verified. This time, uncertainty about the robot displacement, computed from the dead reckoning method, is supplied to the 3DupIC in order to establish consistent data association and error minimization.

Once again, the importance of accurate robot displacement initialization is evaluated by repeating the test twice from each method, either using the dead reckoning solution to initialize the scan matching procedure, or running the registration without supplying an initial displacement estimate.

**Figure 6:**
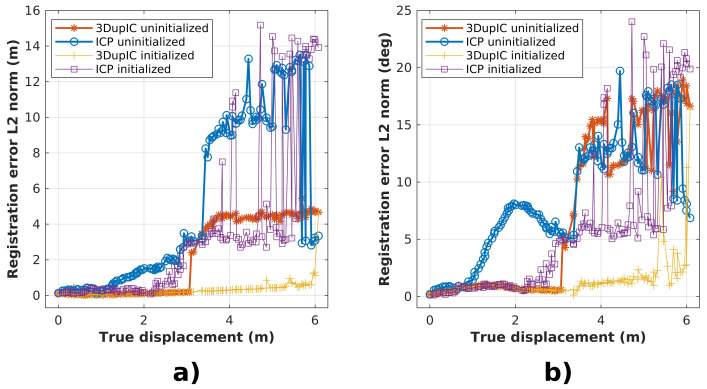
Results of the short trajectory experiment, to evaluate the impact of scan distance on the registration accuracy. The horizontal axis represents the true distance between scan acquisition poses. In the vertical axis, (**a**) displays the position error and (**b**) shows the orientation error.

Results are compared with the ground truth to compute the registration errors, shown in Figure 6. Ideally, the registration algorithms should recover the robot displacement between the scan acquisition poses, but in practice, Figure 6 shows that as the robot displacement increases and scan overlap diminishes, the registration problem becomes harder and divergence occurs at some point. In this particular aspect, the 3DupIC demonstrates the capability to converge for longer relative scan displacements. Moreover, it can be verified that proper initialization delays divergence of both methods. Moreover, the 3DupIC provides the most accurate registration results, with satisfactory accuracy up to 5 m of displacement, which corresponds to a scan overlap of around 40%.

### 3.3. Long-Term Performance

**Figure 7:**
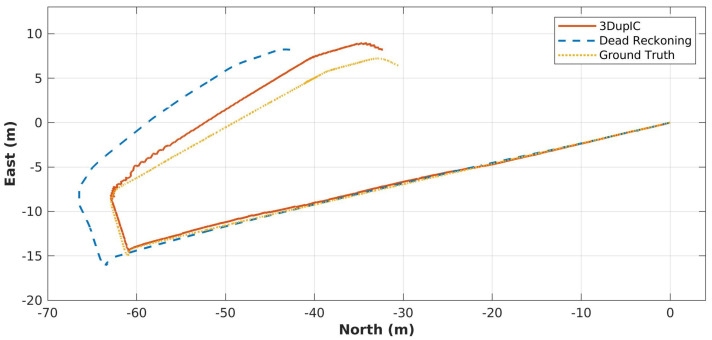
2D representation of the trajectories obtained from dead reckoning (dashed line), from aiding dead reckoning with the 3DupIC corrections (solid line) and the ground truth trajectory (dotted line).

Finally, the transformation sequence obtained by registering consecutive scans, using the 3DupIC, is employed to correct the long-term robot trajectory. 3DupIC corrections are introduced in the dead reckoning’s EKF via a new update, to directly observe position and orientation states. Figure 7 provides a 2D representation of the trajectories obtained by each method, along a robot trajectory of 127 m with a total traveling time of 250 s. It can easily be observed that the integration of 3DupIC solutions contributes positively to reduce dead reckoning drift, as the 3DupIC aided trajectory closely follows the ground truth. At a certain point, around −60 m north and −5 m east, several successive divergences of the 3DupIC are noticeable. They are mainly caused by the flat underwater terrain in that area, which provides insufficient information to support scan matching.

**Figure 8:**
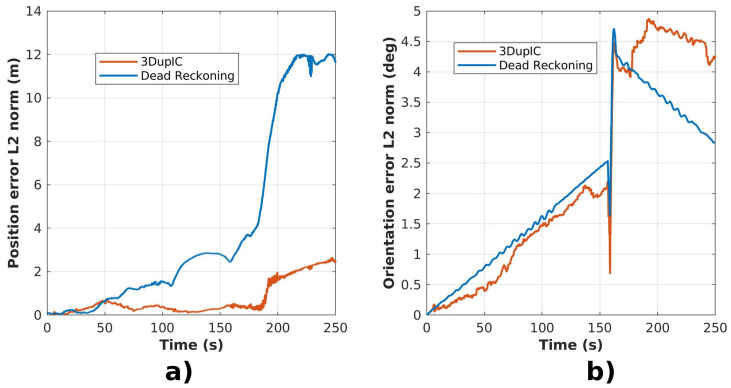
Error comparison between the dead reckoning and the 3DupIC aided dead reckoning solutions. In (**a**), the position error is represented as the Euclidean distance with respect to the ground truth. Similarly, plot (**b**) contains the orientation errors.

A more detailed analysis is presented in Figure 8, where the position and orientation errors of the dead reckoning and the 3DupIC aided solutions are taken with respect to the ground truth. Once again, it can be verified that the 3DupIC aided localization characterizes by much lower position error when compared with the dead reckoning. The performance could be improved further by simply rejecting wrong 3DupIC solutions, as the one that occurs around timestamp 190 s, with great impact in position error. However, we decided to include all 3DupIC updates, without performing any outlier rejection, to provide a more faithful characterization of the algorithm’s performance.

In terms of orientation, at first glance, the impact of 3DupIC contribution is not so striking, since the error follows the dead reckoning tendency, as demonstrated in Figure 8b. Nonetheless, until the robot changes direction, around timestamp 160 s, the 3DupIC presents a slight lower error signature than the dead reckoning. After inverting the direction of motion, despite the higher error of the 3DupIC solution, we can argue that it is more stable than the dead reckoning, and hence more consistent, indicating the ability of the registration method to track the robot orientation with higher precision.

Additionally, we also believe the orientation estimation is being perturbed by a combination of hidden factors, namely inaccurate reference frame calibrations and time synchronization problems between the Echoscope 3D and the remaining sensors. This belief is supported by the presence of an artifact around timestamp 160, when the vehicle changes direction rapidly. At this moment, the orientation error shows two spikes, which indicates poor synchronization of the Echoscope 3D data with respect to the ground truth. On another hand, the transformation from the Echoscope’s sensor frame to the body frame was obtained directly from the robot’s CAD model. Moreover, considering the physical mountings for the Echoscope 3D in the robot’s body, a misalignment of some degrees may occur, leading to poor orientation determination through scan matching.

## 4. Conclusions

This article presents the 3DupIC method for registration of sonar scans, acquired by an Echoscope 3D profiling sonar. The 3DupIC develops a probabilistic scan representation, which enables consistent point matching and error minimization using the Mahalanobis distance. Results demonstrate the superiority of our method when compared with the ICP algorithm. Even when inaccurate initial guesses are supplied, the 3DupIC repeatedly converges to a similar result, close to the real solution. Moreover, our method successfully retrieves a consistent solution in situations of substantial sensor rotation and reduced scan overlap. Additionally, scan matching builds directly on the raw data, without the need for previous scan filtering or subsequent outlier rejection of wrong point matches.

An initial robot displacement, with corresponding uncertainty, obtained from dead reckoning, is supplied to the 3DupIC to facilitate convergence. Results show that our scan matching method consistently refines the initial transformation, so that scan matching updates inserted in the dead reckoning filter produce an improved robot trajectory, with reduced drift.

The experiments reported in this article validate the 3DupIC concept, confirming the feasibility of underwater scan matching in six degrees-of-freedom and its benefit for robot localization. However, before integrating the 3DupIC in a real robot, some additional improvements are necessary. First, the high computational complexity invalidates real-time execution, thus a simplified implementation is necessary. Further, as results indicate, the development of an extrinsic calibration procedure is necessary to refine reference frame transformations, especially to improve the consistency of the 3DupIC derived orientation references. Finally, the calculation of the registration uncertainty is also fundamental to achieve consistent data fusion within the localization estimation framework. For this reason, the determination of the registration covariance matrix should be addressed.

## Data Availability

Not applicable.

## Abbreviations

The following abbreviations are used in this manuscript:

2D	Two-dimensional
3D	Three-dimensional
3DupIC	3D underwater probabilistic Iterative Correspondence
3D	Three-dimensional
pIC	probabilistic Iterative Correspondence
ICP	Iterative Closest Point
AUV	Autonomous Underwater Vehicle
GNSS	Global Navigation Satellite System
MSIS	Mechanical scanning imaging sonar
ICNN	Individual Compatibility Nearest Neighbor
EKF	Extended Kalman Filter
FOG	Fiber Optic Gyroscope
DVL	Doppler Velocity Log
IMU	Inertial Motion Unit
USBL	Ultra-short baseline
SBL	short baseline
PCL	Point Cloud Library
CAD	Computer-aided design
GPU	Graphics Processing Unit
OpenCL	Open Computing Language
RANSAC	Random Sample Consensus
